# Security–Reliability Analysis of AF Full-Duplex Relay Networks Using Self-Energy Recycling and Deep Neural Networks

**DOI:** 10.3390/s23177618

**Published:** 2023-09-02

**Authors:** Tan N. Nguyen, Bui Vu Minh, Dinh-Hieu Tran, Thanh-Lanh Le, Anh-Tu Le, Quang-Sang Nguyen, Byung Moo Lee

**Affiliations:** 1 Communication and Signal Processing Research Group, Faculty of Electrical and Electronics Engineering, Ton Duc Thang University, Ho Chi Minh City 70000, Vietnam; nguyennhattan@tdtu.edu.vn; 2 Faculty of Engineering and Technology, Nguyen Tat Thanh University, 300A-Nguyen Tat Thanh, Ward 13, District 4, Ho Chi Minh City 754000, Vietnam; 3 Department of Technology, Dong Nai Technology University, Bien Hoa 76000, Vietnam; tdh@dntu.edu.vn (D.-H.T.); lethanhlanh@dntu.edu.vn (T.-L.L.); 4 Faculty of Electrical Engineering and Computer Science, VSB-Technical University of Ostrava, 17. Listopadu 2172/15, 70800 Ostrava, Czech Republic; tu.le.anh.st@vsb.cz; 5 Science and Technology Application for Sustainable Development Research Group, Ho Chi Minh City University of Transport, Ho Chi Minh City 70000, Vietnam; sang.nguyen@ut.edu.vn; 6 Department of Intelligent Mechatronics Engineering, and Convergence Engineering for Intelligent Drone, Sejong University, Seoul 05006, Republic of Korea

**Keywords:** physical layer security (PLS), self-energy recycling, full duplex (FD), outage probability (OP), intercept probability (IP), deep learning network (DNN)

## Abstract

This paper investigates the security–reliability of simultaneous wireless information and power transfer (SWIPT)-assisted amplify-and-forward (AF) full-duplex (FD) relay networks. In practice, an AF-FD relay harvests energy from the source (S) using the power-splitting (PS) protocol. We propose an analysis of the related reliability and security by deriving closed-form formulas for outage probability (OP) and intercept probability (IP). The next contribution of this research is an asymptotic analysis of OP and IP, which was generated to obtain more insight into important system parameters. We validate the analytical formulas and analyze the impact on the key system parameters using Monte Carlo simulations. Finally, we propose a deep learning network (DNN) with minimal computation complexity and great accuracy for OP and IP predictions. The effects of the system’s primary parameters on OP and IP are examined and described, along with the numerical data.

## 1. Introduction

The Internet of Things (IoT) is the term employed to describe the interconnection of all physical items with the Internet through information sensing devices for the purpose of information exchange, i.e., the way in which physical objects communicate with one another to accomplish intelligent identification and administration. The future beyond 5G (B5G) IoT and massive machine-type communication (mMTC) will face difficult issues due to massively networked smart gadgets [1]. This is mostly due to the varied quality of service (QoS) provided by the enormous number of such devices for 5G-enabled big IoT networks. As a result of the huge IoT, wireless communication networks will face a variety of issues, including fundamental energy consumption, the use of high-frequency resources, and more [2].

Many approaches have been suggested to boost spectral efficiency (SE) performance. Full-duplex (FD) relaying techniques can, among others, roughly quadruple the SE compared to half-duplex (HD) relaying [3,4,5,6,7]. Additionally, the authors in [8] used orthogonal frequency division multiple access (OFDMA) technology to expand FD relaying into a multi-user scenario. Recent developments in antenna and transceiver design in FD have demonstrated a high potential for eliminating the self-interference (SI) channel up to the receiver noise floor [9]. By utilizing the physical isolation and separation of the transmitter and receiver, the SI channel can lessen passive cancelation. The SI signal in the received transmission is actively suppressed [10]. The FD relay broadcasts information, and the broadcast characteristics of the wireless medium offer a tremendous problem in guaranteeing secure and reliable communications in the face of adversaries [11,12]. As a result, secure transmission becomes a critical problem that cannot be overlooked. Several transmission techniques for enhancing the secrecy rate were presented [13,14,15,16] to prevent secret communications from being eavesdropped in FD relay networks. In [17], the authors investigated a communication network in which a source seeks to interact with an FD destination while being overheard by an eavesdropper. The author in [18] studied the secrecy outage probability (SOP) of the multiple FD decode and forward (DF) relay networks under imperfect channel state information (CSI). In this case, relay selection was applied, which proved to be better than the HD-based strategy. An overview of physical layer security (PLS) schemes for FD co-operative systems was presented in [19]. Furthermore, in a situation with untrusted relays, a source-based jamming strategy was presented, in which the source sends a composite signal comprising the secret and jamming signals to increase secrecy. The authors in [20] examined the scenarios involving different relays and the effects of antenna designs and jamming signals on security. In [21], the authors presented the SOP of an FD jamming relay method, where the source sends data to the relay while sending the jamming signals to the eavesdroppers. Furthermore, the authors in [22] studied a two-hop FD-DF relaying scheme with secrecy rates and optimal power allocation. Finally, Moya. et al. proposed a co-operative network where the FD destination transmits the jamming signal to several amplify-and-forward (AF) untrusted relays in [23].

Energy harvesting (EH) is a workable solution to the problem of limited operation time [24,25,26,27,28,29]. EH can extend the life of the IoT network or even make it self-sufficient by harvesting energy from the environment, such as vibration, solar, and wind [30]. Since it can harvest energy from radio frequency (RF) signals, providing a more reliable energy supply, wireless power transfer (WPT) offers a more realistic solution to the limited period of operation problem [31,32,33]. Researchers have further incorporated the WPT properties into wireless communication systems, known as simultaneous wireless information and power transfer (SWIPT), by taking into account the fact that RF signals may transport both information and energy [34,35,36]. For instance, Chen et al., in [37], investigated limited feedback multi-antenna systems, wherein the trade-off between wireless energy and information transfer was considered. In addition, the authors maximized energy harvesting by using adaptive energy beamforming according to instantaneous CSI. The two protocols for EH, which are time switching (TS) and power splitting (PS), were explored in [38,39], respectively. A part of the time or power of the received signal is utilized for energy harvesting in TS or PS protocols, whereas the remainder is used for information processing. The PLS in the SWIPT network has attracted a lot of researchers, as in [40,41,42]. The secrecy performance of a single-input multiple-output (SIMO) SWIPT system was explored in [40], in which the base station broadcasts information to the receiver while simultaneously transferring energy to numerous energy-harvesting receivers. The authors in [41] presented a strong, secure transmission system for multiple-input single-output (MISO) SWIPT networks. In [42], the authors offered an effective transmission solution for multiple-input multiple-output (MIMO) wiretap channels, in which the non-concave issue was first turned into a convex optimization and then solved by dealing with its dual problem.

Deep learning has recently evolved as a strong data-driven strategy to solve a variety of complex issues, such as image processing, pattern recognition, and wireless communication applications [43]. The authors in [44] designed a deep neural network (DNN) model to forecast coverage probability in random wireless networks. It should be highlighted that the DNN model outperforms the mathematical method, which is only appropriate for oversimplified network settings. Moreover, in [45], the authors employed a DNN model to predict the SOP and demonstrate the shortest running time for SOP prediction across simulation and analytical findings in unmanned aerial vehicle networks. Zheng et al., in [46], studied the combination of adjusted, deep deterministic policy gradient (A-DDPG) and convex optimization to optimize the long-term secondary throughput in RF-powered ambient backscatter-assisted hybrid underlay cognitive radio networks.

### 1.1. Related Work and Motivation

A lot of the current literature has studied the PLS in co-operative relaying networks. In [47], the authors studied the problem of security in untrusted FD relaying using the AF protocol system by applying a source-jamming scheme. However, the author did not consider EH to help improve the lifetime of the device. The authors in [48] investigated security performance in an AF relaying FD system in the presence of a passive eavesdropper. In [49], the authors investigated reliability and security in an AF relaying system in the presence of an eavesdropper. Furthermore, the authors considered friendly jammers to improve the security of the system. However, the authors in [48,49] did not consider FD in co-operative relaying to improve the SE of the system. In addition, the authors of [47,48,49] did not apply a DNN in order to reduce the overall energy consumption through an offline training process. In addition, Table 1 shows a comparison of our work with related works.

Motivated by the challenges described above, we consider the security–reliability of a SWIPT-assisted FD relay in IoT networks. The FD relay harvests energy based on the PS protocol. In particular, the FD relay is also assumed to have the ability for self-energy recycling to increase the average transmittance power. In addition to harvesting the energy from the source’s broadcast signal, the self-interference energy can be recovered and reused [50]. In addition, we apply the DNN method to predict the security–reliability of the proposed system.

### 1.2. Contributions

The main contributions of this paper are listed as follows:

- We propose a novel SWIPT-assisted AF-FD relay network to evaluate security and reliability trade-offs. In particular, in order to increase EH, the relay can harvest energy from the source and reuse the self-interference channel based on the PS protocol to attain battery-free operation;
- We derive the approximate OP for legitimate communications and the approximate IP for the eavesdropper’s channel. The asymptotic expressions for the OP and IP are also examined to give some insight into the system configuration under consideration. In order to verify the derived expressions, Monte-Carlo simulation is adopted;
- The suggested DNN performs almost as well as the simulation while drastically lowering the computing complexity. In comparison to existing machine learning-based regression models for OP/IP prediction, our suggested DNN technique has the lowest root mean square error (RMSE) and takes the shortest time to execute. When system attributes and channel circumstances vary, the data rate of the considered system can be customized based on the estimated OP/IP.

### 1.3. Organization

Section 2 describes the system model. Section 3 expresses the performance analysis. Section 4 shows the asymptotic analysis. Section 5 proposes the DNN network. Section 6 presents numerical results. Finally, a conclusion for the obtained results is presented in Section 7.

## 2. System Model

**Figure 1:**
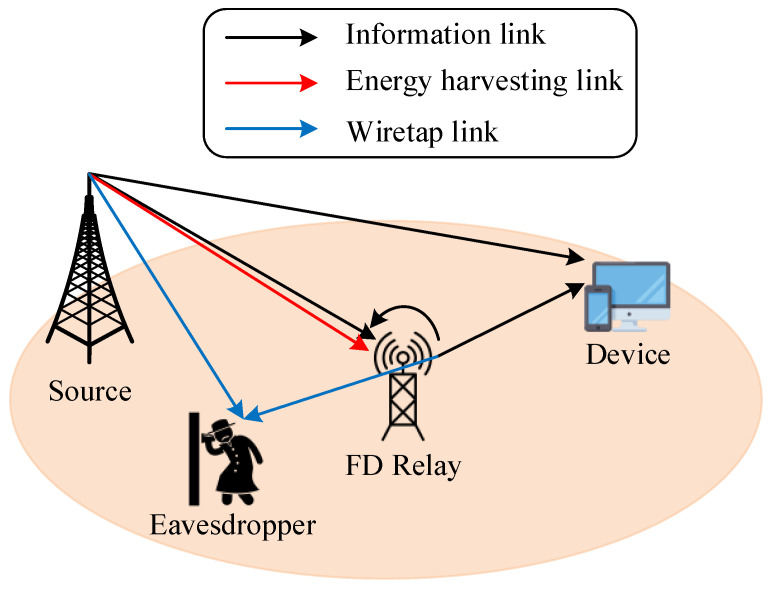
System model.

The proposed system model for wireless communication, in which one source node, S, communicates with one destination node, D, via the help of one FD relay node, R, in the presence of an eavesdropper node, E, that wants to take the information from both R and S is shown in Figure 1. In order to enhance the performance at D, we assumed that S could transmit its signal directly to D and via the relay R. Because of the limited energy, R will need to harvest wireless energy from S and employ the self-energy recycling technique, as seen in [50], and then adopt the total harvested energy to transmit the source data to D using the AF mode. In the AF mode, R amplifies and then forwards the received signal from S to D. Moreover, Table 2 shows the main parameters of our paper.

### 2.1. Energy Harvesting Model

In the energy harvesting phase, in order to implement self-energy recycling (S-ER), the total harvested energy at R can be expressed as [50]

(1) $$\begin{array}{c} E_{R}=\eta \rho T\left(P_{S}\gamma_{\mathrm{SR}}+P_{R}\gamma_{\mathrm{RR}}\right). \end{array}$$

Then, the transmit power of R can be formulated as

(2) $$\begin{array}{c} P_{R}=\frac{E_{R}}{T}=\frac{\eta \rho P_{S}\gamma_{\mathrm{SR}}}{1\;-\;\eta \rho \gamma_{\mathrm{RR}}}. \end{array}$$

It is worth noting from (2) that $P_{R}=0$ when $\gamma_{\mathrm{RR}}\geq \frac{1}{\eta \rho }$. In practice, $\gamma_{\mathrm{RR}}$ is much less than 1 due to passive interference cancellation (IC), such as from antenna isolation, so the denominator in (2) is positive [52].

### 2.2. Fading Channel Model

Let us denote $h_{\mathrm{SD}}$, $h_{\mathrm{SR}}$, $h_{\mathrm{SE}}$, $h_{\mathrm{RD}}$, and $h_{\mathrm{RE}}$ as the channel coefficients of the direct link from source node S to destination node D, and S→R,S→E,R→D,R→E links, respectively. We also denote $h_{\mathrm{RR}}$ as the self-interference coefficient between the transmit and receive antennas of relay node R. Assume that $h_{X}$$\left(X\in \left\{\mathrm{SD},\mathrm{SR},\mathrm{SE},\mathrm{RD},\mathrm{RE}\right\}\right)$ are Rayleigh fading channels; channel gains $\gamma_{X}=|h_{X}{|}^{2}$ are exponential random variables (RVs) for which the cumulative distribution function (CDF) is given as

(3) $$\begin{array}{c} F_{\gamma_{X}}\left(x\right)=1-\exp\left(-\lambda_{X}x\right). \end{array}$$

To take into account the simple path loss model, the parameters can be formulated as follows:

(4) $$\begin{array}{c} \lambda_{X}={\left(d_{X}\right)}^{\omega }. \end{array}$$

The RV $h_{\mathrm{RR}}$ is also modeled as complex Gaussian RV, and hence $\gamma_{\mathrm{RR}}=|h_{\mathrm{RR}}{|}^{2}$ is also an exponential RV. Then, its CDF is given by

(5) $$\begin{array}{c} F_{\gamma_{\mathrm{RR}}}\left(x\right)=1-\exp\left(-\lambda_{\mathrm{RR}}x\right). \end{array}$$

Then, the probability density function (PDF) of $\gamma_{Y}$ is given by

(6) $$\begin{array}{c} f_{\gamma_{Y}}\left(x\right)=\xi \exp\left(-\xi x\right), \end{array}$$

where $\xi \in \left\{\lambda_{\mathrm{SR}},\lambda_{\mathrm{SD}},\lambda_{\mathrm{RD}},\lambda_{\mathrm{SE}},\lambda_{\mathrm{RE}},\lambda_{\mathrm{RR}}\right\}.$

### 2.3. Transmission Model

In the information transmission phase, the received signal at R is given as follows:

(7) $$\begin{array}{c} y_{R}=\sqrt{1-\rho }h_{\mathrm{SR}}x_{S}+\sqrt{1-\rho }h_{\mathrm{RR}}x_{R}+n_{R}. \end{array}$$

Moreover, in this phase, the received signal at D and E are respectively given by:

(8) $$\begin{array}{cc} y_{D}^{1} & =h_{\mathrm{RD}}x_{R}+n_{D}^{1}, \end{array}$$

(9) $$\begin{array}{cc} y_{E}^{1} & =h_{\mathrm{RE}}x_{R}+n_{E}^{1}. \end{array}$$

In our proposed system, the AF protocol is applied. Hence, after receiving the information from S, R will amplify this information to D and E by the given amplification factor β, as follows:

(10) $$\begin{array}{c} \beta =\frac{x_{R}}{y_{R}}=\sqrt{\frac{E\left\{{\left|x_{R}\right|}^{2}\right\}}{E\left\{{\left|y_{R}\right|}^{2}\right\}}}=\sqrt{\frac{P_{R}}{\left(1\;-\;\rho \right)\gamma_{\mathrm{SR}}P_{S}\;+\;\left(1\;-\;\rho \right)\gamma_{\mathrm{RR}}P_{R}\;+\;N_{0}}}. \end{array}$$

By combining (7), (8), (9), and (10), we obtain the received signal at D and E as follows:

(11) $$\begin{array}{cc} y_{D}^{1} & =h_{\mathrm{RD}}\beta \left[\sqrt{1-\rho }h_{\mathrm{SR}}x_{S}+\sqrt{1-\rho }h_{\mathrm{RR}}x_{R}+n_{R}\right]+n_{D}^{1}\\ \; & =\underset{signal}{\underset{︸}{h_{\mathrm{RD}}\beta \sqrt{1-\rho }h_{\mathrm{SR}}x_{S}}}+\underset{interference}{\underset{︸}{h_{\mathrm{RD}}\beta \sqrt{1-\rho }h_{\mathrm{RR}}x_{R}}}+\underset{noise}{\underset{︸}{h_{\mathrm{RD}}\beta n_{R}+n_{D}^{1}}}, \end{array}$$

and

(12) $$\begin{array}{c} y_{E}^{1}=\underset{signal}{\underset{︸}{h_{\mathrm{RE}}\beta \sqrt{1-\rho }h_{\mathrm{SR}}x_{S}}}+\underset{interference}{\underset{︸}{h_{\mathrm{RE}}\beta \sqrt{1-\rho }h_{\mathrm{RR}}x_{R}}}+\underset{noise}{\underset{︸}{h_{\mathrm{RE}}\beta n_{R}+n_{E}^{1}}}. \end{array}$$

The received signal-to-interference plus noise ratio (SINR) at D and E in this phase can be, thus, calculated using the following expressions:

(13) $$\begin{array}{c} \gamma_{D}^{1}=\frac{E\left\{{\left|signal\right|}^{2}\right\}}{E\left\{{\left|noise\right|}^{2}\right\}}=\frac{\gamma_{\mathrm{SR}}\gamma_{\mathrm{RD}}\beta^{2}\left(1\;-\;\rho \right)P_{S}}{\gamma_{\mathrm{RR}}\gamma_{\mathrm{RD}}\beta^{2}\left(1\;-\;\rho \right)P_{R}\;+\;\gamma_{\mathrm{RD}}\beta^{2}N_{0}\;+\;N_{0}}, \end{array}$$

and

(14) $$\begin{array}{c} \gamma_{E}^{1}=\frac{E\left\{{\left|signal\right|}^{2}\right\}}{E\left\{{\left|noise\right|}^{2}\right\}}=\frac{\gamma_{\mathrm{SR}}\gamma_{\mathrm{RE}}\beta^{2}\left(1\;-\;\rho \right)P_{S}}{\gamma_{\mathrm{RR}}\gamma_{\mathrm{RE}}\beta^{2}\left(1\;-\;\rho \right)P_{R}\;+\;\gamma_{\mathrm{RE}}\beta^{2}N_{0}\;+\;N_{0}}. \end{array}$$

By substituting (2) into (13) and (14) and then carrying out some algebra, the SINR at D and E can be rewritten as

(15) $$\gamma_{D}^{1}=\frac{\gamma_{\mathrm{SR}}\gamma_{\mathrm{RD}}\eta \rho \left(1-\eta \rho \gamma_{\mathrm{RR}}\right)\Psi }{\gamma_{\mathrm{SR}}\gamma_{\mathrm{RD}}\eta^{2}\rho^{2}\Psi \gamma_{\mathrm{RR}}-\eta \rho \gamma_{\mathrm{RR}}+1},$$

(16) $$\gamma_{E}^{1}=\frac{\gamma_{\mathrm{SR}}\gamma_{\mathrm{RE}}\eta \rho \left(1-\eta \rho \gamma_{\mathrm{RR}}\right)\Psi }{\gamma_{\mathrm{SR}}\gamma_{\mathrm{RE}}\eta^{2}\rho^{2}\Psi \gamma_{\mathrm{RR}}-\eta \rho \gamma_{\mathrm{RR}}+1},$$

where $\Psi =\frac{P_{S}}{N_{0}}$ denotes the average transmited signal-to-noise ratio (SNR).

In our proposed model, the direct link is considered. Hence, in the broadcast phase, D can be received, and the direct signal from S and E can overhear this signal when S broadcasts to R and D. As a result, the received signal at D and E can be thus expressed by

(17) $$\begin{array}{cc} y_{D}^{2} & =h_{\mathrm{SD}}x_{S}+n_{D}^{2}, \end{array}$$

(18) $$\begin{array}{cc} y_{E}^{2} & =h_{\mathrm{SE}}x_{S}+n_{E}^{2}. \end{array}$$

The SNR at D and E in this phase can be computed respectively by

(19) $$\begin{array}{cc} \gamma_{D}^{2} & =\Psi \gamma_{\mathrm{SD}}, \end{array}$$

(20) $$\begin{array}{cc} \gamma_{E}^{2} & =\Psi \gamma_{\mathrm{SE}}. \end{array}$$

Finally, by adopting the selection-combining (SC) technique at the receiver, the end-to-end SNR at D and E can be respectively claimed as

(21) $$\begin{array}{cc} \gamma_{D} & =\max\left(\gamma_{D}^{1},\gamma_{D}^{2}\right), \end{array}$$

(22) $$\begin{array}{cc} \gamma_{E} & =\max\left(\gamma_{E}^{1},\gamma_{E}^{2}\right). \end{array}$$

## 3. Performance Analysis

In this section, the performance of the proposed system is studied. In particular, the closed-form outage probability (OP) and intercept probability (IP) are derived.

### 3.1. Outage Probability Analysis

The OP of the system can be expressed by

(23) $$\begin{array}{c} \mathrm{OP}=\mathrm{Pr}\left(\gamma_{D}⩽\gamma_{\mathrm{th}}\right), \end{array}$$

where $\gamma_{\mathrm{th}}=2^{R_{\mathrm{th}}}-1$ is the threshold, and $R_{\mathrm{th}}$ is the target rate. From (21) and (23), the OP can be rewritten as

(24) $$\begin{array}{c} \mathrm{OP}=\mathrm{Pr}\left(\max\left(\gamma_{D}^{1},\gamma_{D}^{2}\right)⩽\gamma_{\mathrm{th}}\right)\\ =\mathrm{Pr}\left(\max\left(\frac{\gamma_{\mathrm{SR}}\gamma_{\mathrm{RD}}\eta \rho \left(1\;-\;\eta \rho \gamma_{\mathrm{RR}}\right)\Psi }{\gamma_{\mathrm{SR}}\gamma_{\mathrm{RD}}\eta^{2}\rho^{2}\Psi \gamma_{\mathrm{RR}}\;-\;\eta \rho \gamma_{\mathrm{RR}}\;+\;1},\Psi \gamma_{\mathrm{SD}}\right)⩽\gamma_{\mathrm{th}}\right)\\ =\underset{\Upsilon_{1}}{\underset{︸}{\mathrm{Pr}\left(\Psi \gamma_{\mathrm{SD}}⩽\gamma_{\mathrm{th}}\right)}}\underset{\Upsilon_{2}}{\underset{︸}{\mathrm{Pr}\left(\frac{\gamma_{\mathrm{SR}}\gamma_{\mathrm{RD}}\eta \rho \left(1\;-\;\eta \rho \gamma_{\mathrm{RR}}\right)\Psi }{\gamma_{\mathrm{SR}}\gamma_{\mathrm{RD}}\eta^{2}\rho^{2}\Psi \gamma_{\mathrm{RR}}\;-\;\eta \rho \gamma_{\mathrm{RR}}\;+\;1}⩽\gamma_{\mathrm{th}}\right)}}. \end{array}$$

Based on (24), $\Upsilon_{1}$ can be figured out as

(25) $$\begin{array}{c} \Upsilon_{1}=\mathrm{Pr}\left(\Psi \gamma_{\mathrm{SD}}⩽\gamma_{\mathrm{th}}\right)=\mathrm{Pr}\left(\gamma_{\mathrm{SD}}⩽\frac{\gamma_{\mathrm{th}}}{\Psi }\right)=1-\exp\left(-\frac{\lambda_{\mathrm{SD}}\gamma_{\mathrm{th}}}{\Psi }\right). \end{array}$$

Next, $\Upsilon_{2}$ can be, thus, computed by

(26) $$\begin{array}{c} \Upsilon_{2}=\mathrm{Pr}\left(\frac{\gamma_{\mathrm{SR}}\gamma_{\mathrm{RD}}\eta \rho \left(1\;-\;\eta \rho \gamma_{\mathrm{RR}}\right)\Psi }{\gamma_{\mathrm{SR}}\gamma_{\mathrm{RD}}\eta^{2}\rho^{2}\Psi \gamma_{\mathrm{RR}}\;-\;\eta \rho \gamma_{\mathrm{RR}}\;+\;1}⩽\gamma_{\mathrm{th}}\right)\\ =\mathrm{Pr}\left(\gamma_{\mathrm{SRD}}<\frac{\gamma_{\mathrm{th}}\left(1\;-\;\eta \rho \gamma_{\mathrm{RR}}\right)}{\eta \rho \left(1\;-\;\eta \rho \gamma_{\mathrm{RR}}\right)\Psi \;-\;\gamma_{\mathrm{th}}\eta^{2}\rho^{2}\Psi \gamma_{\mathrm{RR}}}\right), \end{array}$$

where $\gamma_{\mathrm{SRD}}=\gamma_{\mathrm{SR}}\gamma_{\mathrm{RD}}$. From (26), there are two cases to calculate $\Upsilon_{2}$. In the first case, when $\gamma_{\mathrm{RR}}⩽\frac{1}{\eta \rho \left(1\;+\;\gamma_{\mathrm{th}}\right)}$, we obtain $\Upsilon_{2}=\mathrm{Pr}\left(\gamma_{\mathrm{SRD}}<\frac{\gamma_{\mathrm{th}}\left(1\;-\;\eta \rho \gamma_{\mathrm{RR}}\right)}{\eta \rho \left(1\;-\;\eta \rho \gamma_{\mathrm{RR}}\right)\Psi \;-\;\gamma_{\mathrm{th}}\eta^{2}\rho^{2}\Psi \gamma_{\mathrm{RR}}}\right)$. In the second case, when $\gamma_{\mathrm{RR}}>\frac{1}{\eta \rho \left(1\;+\;\gamma_{\mathrm{th}}\right)}$, we obtain $\Upsilon_{2}=1$. Then, in case $\gamma_{\mathrm{RR}}⩽\frac{1}{\eta \rho \left(1\;+\;\gamma_{\mathrm{th}}\right)}$, $\Upsilon_{2}$ can be calculated as

(27) $$\begin{array}{c} \Upsilon_{2}=\int_{\frac{1}{\eta \rho \left(1\;+\;\gamma_{\mathrm{th}}\right)}}^{+\infty }f_{\gamma_{\mathrm{RR}}}\left(y\right)dy+\int_{0}^{\frac{1}{\eta \rho \left(1\;+\;\gamma_{\mathrm{th}}\right)}}F_{\gamma_{\mathrm{SRD}}}\left[\frac{\gamma_{\mathrm{th}}\left(1\;-\;\eta \rho y\right)}{\eta \rho \left(1\;-\;\eta \rho y\right)\Psi -\gamma_{\mathrm{th}}\eta^{2}\rho^{2}\Psi y}\right]f_{\gamma_{\mathrm{RR}}}\left(y\right)dy. \end{array}$$

In order to find $\Upsilon_{2}$, first, we have to derive the CDF of $\gamma_{\mathrm{SRD}}$. As a result, we claim

(28) $$\begin{array}{c} F_{\gamma_{\mathrm{SRD}}}\left(x\right)=\mathrm{Pr}\left(\gamma_{\mathrm{SRD}}<x\right)=\mathrm{Pr}\left(\gamma_{\mathrm{SR}}<\frac{x}{\gamma_{\mathrm{RD}}}\right)\\ =\int_{0}^{+\infty }F_{\gamma_{\mathrm{SR}}}\left(\frac{x}{y}\right)f_{\gamma_{\mathrm{RD}}}\left(y\right)dy\\ =1-\int_{0}^{+\infty }\lambda_{\mathrm{RD}}\exp\left(-\frac{\lambda_{\mathrm{SR}}x}{y}-\lambda_{\mathrm{RD}}y\right)dy. \end{array}$$

By applying [53] (Eq. 3.324.1), we obtain

(29) $$F_{\gamma_{\mathrm{SRD}}}\left(x\right)=1-2\sqrt{\lambda_{\mathrm{SR}}\lambda_{\mathrm{RD}}x}K_{1}\left(2\sqrt{\lambda_{\mathrm{SR}}\lambda_{\mathrm{RD}}x}\right),$$

where $K_{\nu }\left(•\right)$ is the modified Bessel function of the second kind with ν-th order. From (26) and (29), $\Upsilon_{2}$ can be found as

(30) $$\Upsilon_{2}=1-2\lambda_{\mathrm{RR}}\int_{0}^{\frac{1}{\eta \rho \left(1\;+\;\gamma_{\mathrm{th}}\right)}}\sqrt{\lambda_{\mathrm{SR}}\lambda_{\mathrm{RD}}\Lambda \left(y\right)}\exp\left(-\lambda_{\mathrm{RR}}y\right)K_{1}\left(2\sqrt{\lambda_{\mathrm{SR}}\lambda_{\mathrm{RD}}\Lambda \left(y\right)}\right)dy,$$

where $\Lambda \left(y\right)=\frac{\gamma_{\mathrm{th}}\left(1\;-\;\eta \rho y\right)}{\eta \rho \left(1\;-\;\eta \rho y\right)\Psi \;-\;\gamma_{\mathrm{th}}\eta^{2}\rho^{2}\Psi y}$. Unfortunately, the integral in $\Upsilon_{2}$ presents a tough task in terms of finding a closed-form expression. Therefore, we apply the Gaussian-Chebyshev quadrature in [54] to approximate this. As a result, $\Upsilon_{2}$ can be obtained by

(31) $$\begin{array}{c} \Upsilon_{2}\approx 1\;-\;\frac{\pi \lambda_{\mathrm{RR}}}{N\eta \rho \left(1\;+\;\gamma_{\mathrm{th}}\right)}\sum_{n=1}^{N}\sqrt{1-\varphi_{n}^{2}}\sqrt{\lambda_{\mathrm{SR}}\lambda_{\mathrm{RD}}\Lambda \left(\frac{\left(1\;+\;\varphi_{n}\right)}{2\eta \rho \left(1\;+\;\gamma_{\mathrm{th}}\right)}\right)}\\ \times \exp\left(-\lambda_{\mathrm{RR}}\frac{\left(1\;+\;\varphi_{n}\right)}{2\eta \rho \left(1\;+\;\gamma_{\mathrm{th}}\right)}\right)K_{1}\left(2\sqrt{\lambda_{\mathrm{SR}}\lambda_{\mathrm{RD}}\Lambda \left(\frac{\left(1\;+\;\varphi_{n}\right)}{2\eta \rho \left(1\;+\;\gamma_{\mathrm{th}}\right)}\right)}\right), \end{array}$$

where $\varphi_{n}=\cos\left(\frac{2n\;-\;1}{2N}\pi \right)$. Finally, by substituting (25) and (31) into (23), the OP can be, thus, obtained as

(32) $$\begin{array}{c} \mathrm{OP}\approx \left\{1-\exp\left(-\frac{\lambda_{\mathrm{SD}}\gamma_{\mathrm{th}}}{\Psi }\right)\right\}\left\{1-\frac{\pi \lambda_{\mathrm{RR}}}{N\eta \rho \left(1\;+\;\gamma_{\mathrm{th}}\right)}\sum_{n=1}^{N}\sqrt{1-\varphi_{n}^{2}}\sqrt{\lambda_{\mathrm{SR}}\lambda_{\mathrm{RD}}\Lambda \left(\frac{\left(1\;+\;\varphi_{n}\right)}{2\eta \rho \left(1\;+\;\gamma_{\mathrm{th}}\right)}\right)}\right.\\ \left.\times \exp\left(-\lambda_{\mathrm{RR}}\frac{\left(1\;+\;\varphi_{n}\right)}{2\eta \rho \left(1\;+\;\gamma_{\mathrm{th}}\right)}\right)K_{1}\left(2\sqrt{\lambda_{\mathrm{SR}}\lambda_{\mathrm{RD}}\Lambda \left(\frac{\left(1\;+\;\varphi_{n}\right)}{2\eta \rho \left(1\;+\;\gamma_{\mathrm{th}}\right)}\right)}\right)\right\}. \end{array}$$

### 3.2. Intercept Probability Analysis

The considered system will be wiretapped if E can successfully decode the received signals from the source and relay [55,56]. Therefore, the IP is given by

(33) $$\begin{array}{c} \mathrm{IP}=\mathrm{Pr}\left(\gamma_{E}⩾\gamma_{\mathrm{th}}\right)=1-\mathrm{Pr}\left(\gamma_{E}<\gamma_{\mathrm{th}}\right)\\ =1-\left\{\mathrm{Pr}\left(\Psi \gamma_{\mathrm{SE}}<\gamma_{\mathrm{th}}\right)\right\}\left\{\mathrm{Pr}\left(\frac{\gamma_{\mathrm{SR}}\gamma_{\mathrm{RE}}\eta \rho \left(1\;-\;\eta \rho \gamma_{\mathrm{RR}}\right)\Psi }{\gamma_{\mathrm{SR}}\gamma_{\mathrm{RE}}\eta^{2}\rho^{2}\Psi \gamma_{\mathrm{RR}}\;-\;\eta \rho \gamma_{\mathrm{RR}}\;+\;1}\right)<\gamma_{\mathrm{th}}\right\}. \end{array}$$

As a similar proof for OP, the IP can be achieved by

(34) $$\begin{array}{c} \mathrm{IP}\approx 1-\left\{1-\exp\left(-\frac{\lambda_{\mathrm{SE}}\gamma_{\mathrm{th}}}{\Psi }\right)\right\}\left\{1-\frac{\pi \lambda_{\mathrm{RR}}}{N\eta \rho \left(1\;+\;\gamma_{\mathrm{th}}\right)}\sum_{n=1}^{N}\sqrt{1-\varphi_{n}^{2}}\sqrt{\lambda_{\mathrm{SR}}\lambda \Lambda \left(\frac{\left(1\;+\;\varphi_{n}\right)}{2\eta \rho \left(1\;+\;\gamma_{\mathrm{th}}\right)}\right)}\right.\\ \left.\times \exp\left(-\lambda_{\mathrm{RR}}\frac{\left(1\;+\;\varphi_{n}\right)}{2\eta \rho \left(1\;+\;\gamma_{\mathrm{th}}\right)}\right)K_{1}\left(2\sqrt{{}_{\mathrm{SR}}\lambda_{\mathrm{RE}}\Lambda \left(\frac{\left(1\;+\;\varphi_{n}\right)}{2\eta \rho \left(1\;+\;\gamma_{\mathrm{th}}\right)}\right)}\right)\right\}, \end{array}$$

where $\Lambda \left(y\right)=\frac{\gamma_{\mathrm{th}}\left(1\;-\;\eta \rho y\right)}{\eta \rho \left(1\;-\;\eta \rho y\right)\Psi \;-\;\gamma_{\mathrm{th}}\eta^{2}\rho^{2}\Psi y}$.

## 4. Asymptotic Analysis

In this section, we develop the asymptotic equations for OP as the transmitted SNR approaches infinity, i.e., Ψ→+∞, to give us more insights into the performance analysis of the network under consideration.

### 4.1. Op Analysis

When Ψ→+∞, $\gamma_{D}$ can be rewritten as

(35) $$\begin{array}{c} \gamma_{D}^{\Psi \to +\infty }\approx \max\left(\Psi \gamma_{\mathrm{SD}},\frac{1}{\eta \rho \gamma_{\mathrm{RR}}}-1\right). \end{array}$$

Then, the OP can be obtained by

(36) $$\begin{array}{c} {\mathrm{OP}}^{\Psi \to +\infty }=\mathrm{Pr}\left(\gamma_{D}^{\Psi \to +\infty }<\gamma_{\mathrm{th}}\right)\\ =\mathrm{Pr}\left(\gamma_{\mathrm{SD}}<\frac{\gamma_{\mathrm{th}}}{\Psi }\right)\mathrm{Pr}\left(\gamma_{\mathrm{RR}}>\frac{1}{\eta \rho \left(1\;+\;\gamma_{\mathrm{th}}\right)}\right)\\ =\left[1-\exp\left(-\frac{\lambda_{\mathrm{SD}}\gamma_{\mathrm{th}}}{\Psi }\right)\right]\exp\left(-\frac{\lambda_{\mathrm{RR}}}{\eta \rho \left(1\;+\;\gamma \right)}\right). \end{array}$$

### 4.2. Ip Asymptotic Analysis

As a result, in this case, the IP also can be obtained by

(37) $$\begin{array}{c} {\mathrm{IP}}^{\Psi \to +\infty }=\exp\left(-\frac{\lambda_{\mathrm{SE}}\gamma_{\mathrm{th}}}{\Psi }\right)\left[1-\exp\left(-\frac{\lambda_{\mathrm{RR}}}{\eta \rho \left(1\;+\;\gamma_{\mathrm{th}}\right)}\right)\right]. \end{array}$$

## 5. Dnn Network

In this section, we propose a DNN to predict the OP and IP without relying on the statistical model, whereas the traditional analysis and Monte Carlo simulations need an accurate statistical model. In addition, when the system model is complicated, and it is difficult to use the mathematical derivation technique, the DNN model, which is a data-driven approach, becomes an alternate answer. Therefore, the DNN will help the proposed system to achieve a short run time.

### 5.1. The DNN Design Description

**Figure 2:**
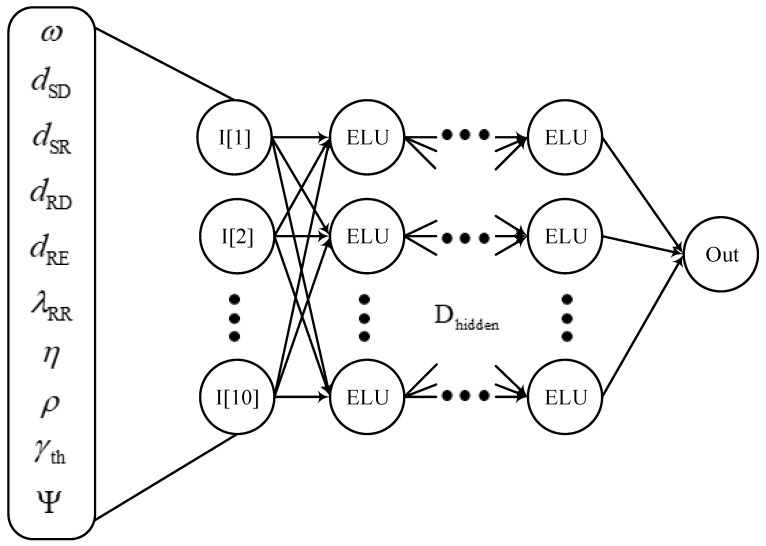
A diagram of the DNN architecture.

First, we create a DNN model as a regression issue. As illustrated in Figure 2, the DNN model consists of an input layer, numerous hidden layers, and an output layer. The following is a summary of how each layer contributes to training the DNN model:

- Data is sent to the input layer so that the DNN model may determine how the system parameters relate to the relevant OP/IP. The number of neurons in the input layer is, therefore, equal to the number of parameters and does not serve as an activation function;
- The number of hidden layers primarily determines the relationship between the input and output data. In order to accurately calculate the relationship, each connection in each hidden neuron has a separate weight and bias. In order to enhance computational effectiveness, each hidden neuron also has a nonlinear activation function;
- The output layer combines the findings of various hidden layers to predict OP/IP. As a result, there is just one neuron in the output layer. The neuron in the output layer lacks an activation function, much like the input layer.

Furthermore, we have 10 neurons corresponding to 10 parameters, as shown in Table 3 for the input layer. In the hidden layers, each layer *k* with $k=1,\ldots ,D_{\mathrm{hidden}}$ has $D_{\mathrm{neu}}$ neurons, and it employs the exponential linear unit (ELU) activation function, which can be given as [57,58]

(38) $$\begin{array}{c} \mathrm{ELU}\left(z\right)=\left\{\begin{array}{c} \varphi (\exp(z)-1),\;\mathrm{If}:\;z<0\\ z,\;\;\;\;\;\;\;\mathrm{If}:\;z⩾0 \end{array}\right. \end{array}$$

where φ denotes the constant value initialized to 1. Since the regression problem tries to estimate an output value without additional conversion, the output layer comprises one neuron that uses the linear activation function to produce the predicted OP/IP value, Out.

### 5.2. Dataset Setup

In this subsection, we generate dataset D as a row vector for each sample *i*, i.e., Data $\left[k\right]=[I\left[k\right],{\mathrm{Out}}_{\mathrm{Sim}}]$, where I[k] is the feature vector containing all the inputs from the parameters listed in Table 3. Each feature I[k] is utilized to produce real-value OP/IP sets from (23) and (33); this is input into the simulation, and a unique matching ${\mathrm{Out}}_{\mathrm{Sim}}$ is returned. In conclusion, we built the dataset by generating $10^{5}$ samples, concatenating them, and then dividing this into a new dataset with 80% for training $\left(D_{\mathrm{train}}\right)$, 10% for validation $\left(D_{\mathrm{vali}}\right)$, and 10% for testing $\left(D_{\mathrm{test}}\right)$. Moreover, we set the DNN model to have four hidden layers and 128 hidden neurons, which is implemented in Python 3.11.4 using Keras 2.8.0 and TensorFlow 2.8.0. Furthermore, the DNN model is trained in 100 epochs. The deep model is specifically constructed using hardware with an AMDRyzen Threadripper 3970X 32-core CPU and an Nvidia GeForce RTX-2070 super GPU for rapid training and experiment simulations.

The estimation accuracy of the DNN model is calculated using the mean-square error (MSE), which is formally stated as $\mathrm{MSE}=\frac{1}{D_{\mathrm{test}}}\sum_{k=0}^{D_{\mathrm{test}}-1}\left[{\mathrm{Out}}_{\mathrm{Pre}}-{\mathrm{Out}}_{\mathrm{Sim}}\right]$. Furthermore, the appropriate weights and biases for each connection are determined by applying the Adam optimizer [59]. The difference between the natural and predicted OP/IP values throughout the full test set, which is specified as $\mathrm{RMSE}=\sqrt{\mathrm{MSE}}$, is measured by using the RMSE in the OP/IP prediction.

## 6. Numerical Results

In this section, we provide the analysis findings to evaluate the proposed system in terms of OP and IP, as well as the simulation results, by using the Monte Carlo approach, as per [60,61], to validate our analytical derivations. The main parameter can be shown in Table 3, except for some specific cases.

**Figure 3:**
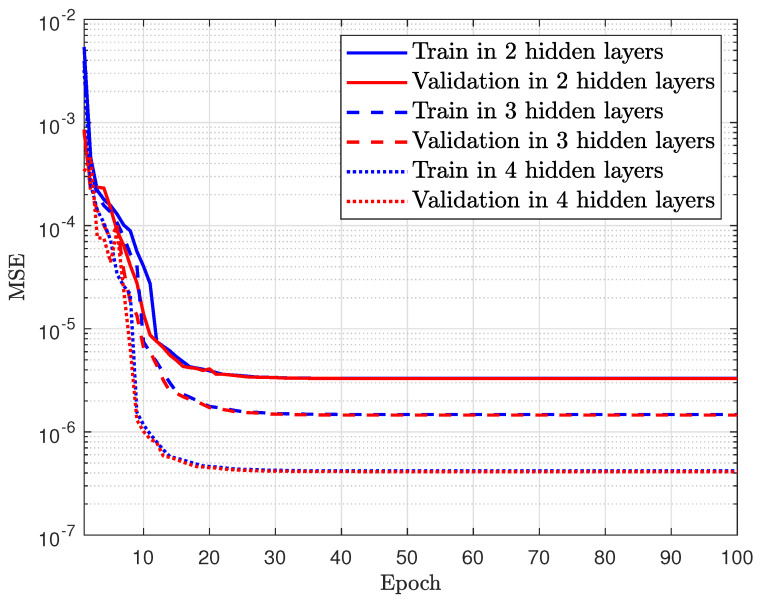
MSE convergence in training and evaluating the DNN with varying the hidden layers.

In Figure 3, we utilize the validation set to evaluate the accuracy of the training. As can be observed, when increasing the epoch and number of hidden layers, the MSE decreased. Moreover, the MSE in the four hidden layers is the best case. Although the DNN model contains four hidden layers that may generalize the dataset and improve network capacity, the second and third hidden layers are unable to learn the intricate patterns in a high-dimensional dataset, resulting in a large MSE.

**Figure 4:**
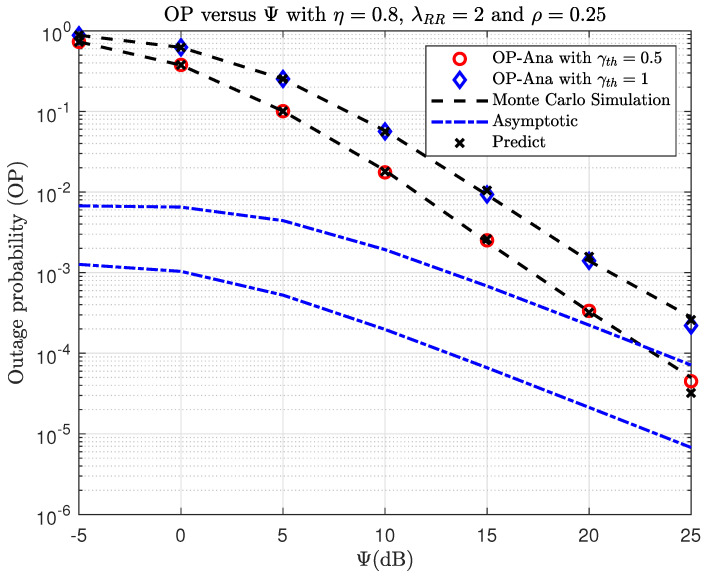
TheOP versus Ψ(dB) when varying $\gamma_{th}$ with η=0.8, $\lambda_{RR}=2$, and ρ=0.25.

**Figure 5:**
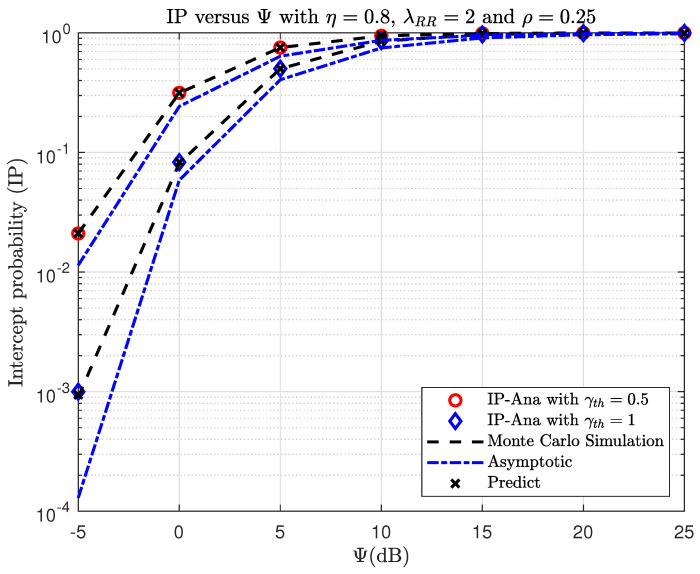
TheIP versus Ψ(dB) when varying $\gamma_{th}$ with η=0.8, $\lambda_{RR}=2$, and ρ=0.25.

Figure 4 and Figure 5 show the OP and IP versus Ψ(dB) with different $\gamma_{th}$. As observed, the OP and IP curves correspond exactly to the Monte Carlo simulation results. By looking at Figure 4, the OP performance decreases if the Ψ increases. When Ψ is large, the SINR will significantly improve, and this will make the OP performance better. In Figure 5, it can be observed that as Ψ increases, the IP performance also increases. This is expected because an eavesdropper is more likely to overhear the message when the transmission power at S is higher. At a high SNR, i.e., Ψ→∞, it can be seen that the asymptotic OP and IP curves closely match the actual findings. Specifically, the IP converges to the asymptotic value when Ψ=15(dB), whereas the OP converges to the asymptotic value at a higher Ψ (Ψ>25(dB), which cannot be seen in Figure 4). In addition, it can be shown that the DNN-based prediction results are very similar to the simulation and analysis results for OP and IP, demonstrating the superior prediction capabilities of the DNN.

**Figure 6:**
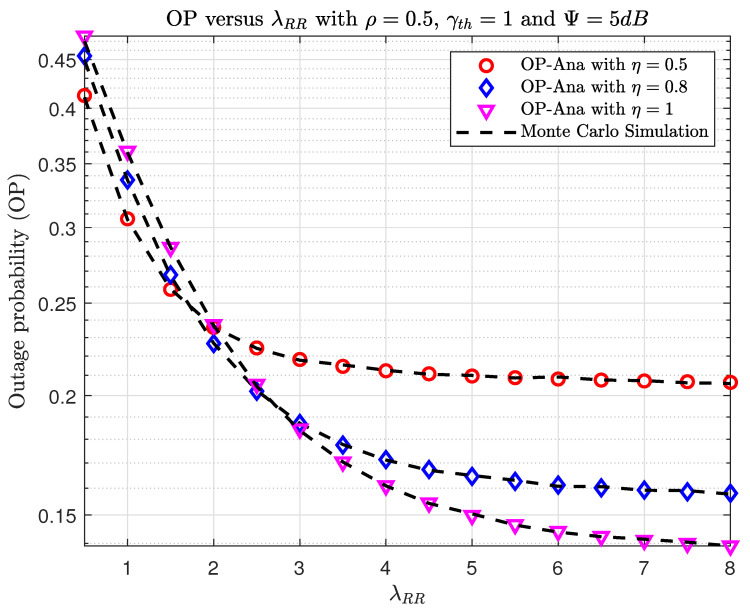
The OP versus $\lambda_{RR}$ when varying η with ρ=0.5, $\gamma_{th}=1$, and Ψ=5(dB).

**Figure 7:**
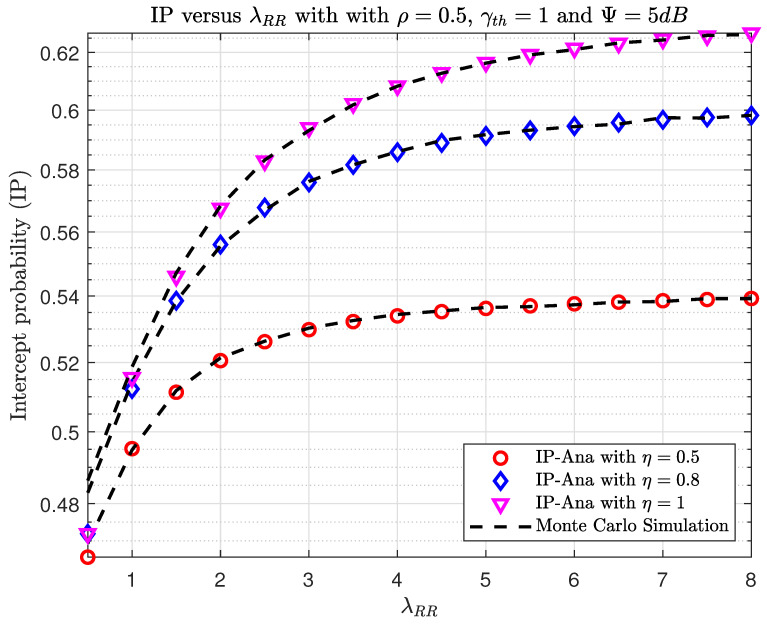
The IP versus $\lambda_{RR}$ when varying η with ρ=0.5, $\gamma_{th}=1$, and Ψ=5(dB).

In Figure 6 and Figure 7, we plot the OP and IP versus $\lambda_{RR}$ with different η. In Figure 6, increasing the $\lambda_{RR}$ between the transmitting and receiving antennas at the relay decreases the OP. It can be explained by the fact that increasing $\lambda_{RR}$ will make the $\gamma_{D}^{1}$ in (15) larger; hence, the OP will be better. Moreover, when increasing energy efficiency η, the average transmit power at R will be higher, and this will then lead to an improvement in OP. Furthermore, when increasing $\lambda_{RR}$ and η, the SINR at E becomes larger. Thus, the possibility of E eavesdropping on information from S and R is also very high. So, the problem is that we have to trade-off between security and reliability in terms of OP and IP. This means that if the system wants to operate well, we must accept high eavesdropping information and vice versa.

**Figure 8:**
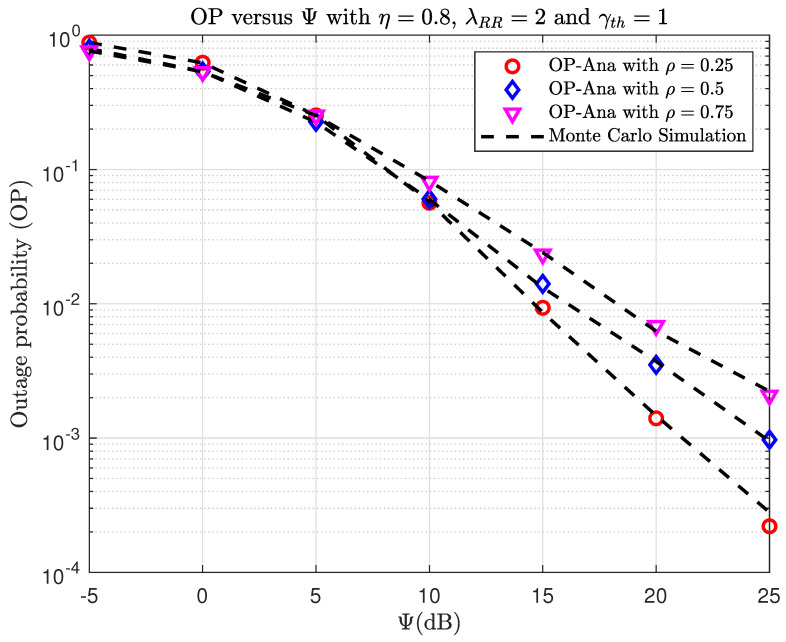
TheOP versus Ψ(dB) when varying ρ with η=0.8, $\lambda_{RR}=2$, and $\gamma_{th}=1$.

**Figure 9:**
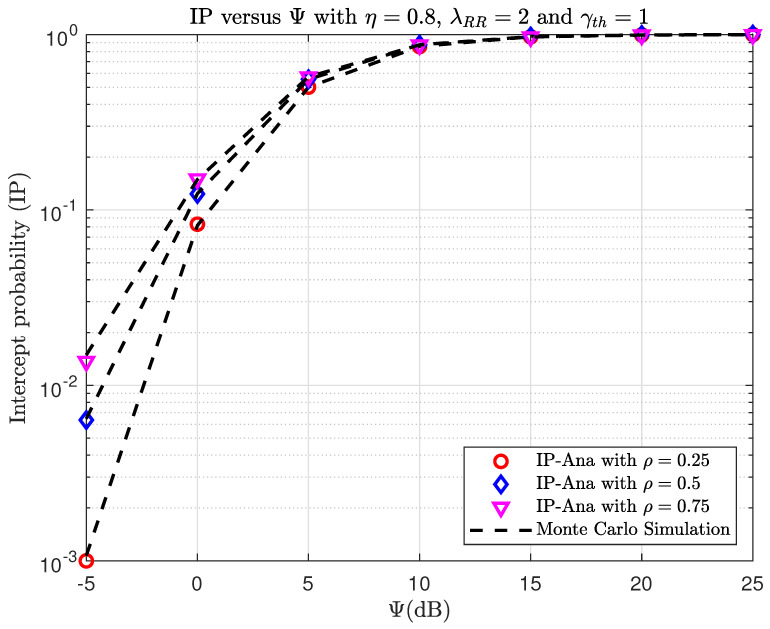
TheIP versus Ψ(dB) when varying ρ with η=0.8, $\lambda_{RR}=2$, and $\gamma_{th}=1$.

In Figure 8 and Figure 9, we plot the OP and IP versus Ψ(dB) with different PS factors, ρ. First, The higher the Ψ value in Figure 8, the better the OP. This is explained by the fact that the higher the Ψ value, the more the transmitted power of source S is assigned. Second, it is easy to observe that the OP decreases when the PS factor increases. Third, it can be seen that for a small Ψ (Ψ<5(dB)), the use of a large PS factor is more beneficial. Reversely, at higher Ψ, the smaller ρ is better. The reason is as follows. For the high-noise environment case, higher transmitted power at the relay is needed to guarantee successful communication. That means more energy needs to be harvested at the relay, so a larger ρ is better. On the other hand, if Ψ is large, then the decoding of the message at the relay is more important. That means we should select the smaller ρ. As can be observed in Figure 9, the intercept performance improves when Ψ increases. This is expected because the eavesdropper has a better chance of overhearing the communication with a greater source transmit power, S. When Ψ is large enough, the IP can converge to one. The eavesdropper’s IP increases as the PS factor increases, which is due to the high transmitted power of relay R.

**Figure 10:**
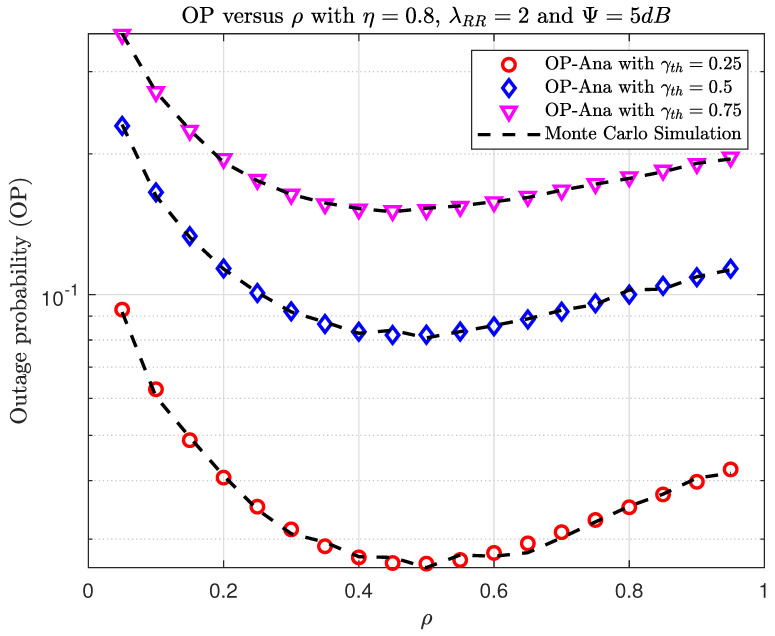
TheOP versus ρ when varying $\gamma_{th}$ with η=0.8, $\lambda_{RR}=2$, and $\Psi_{th}=5$(dB).

**Figure 11:**
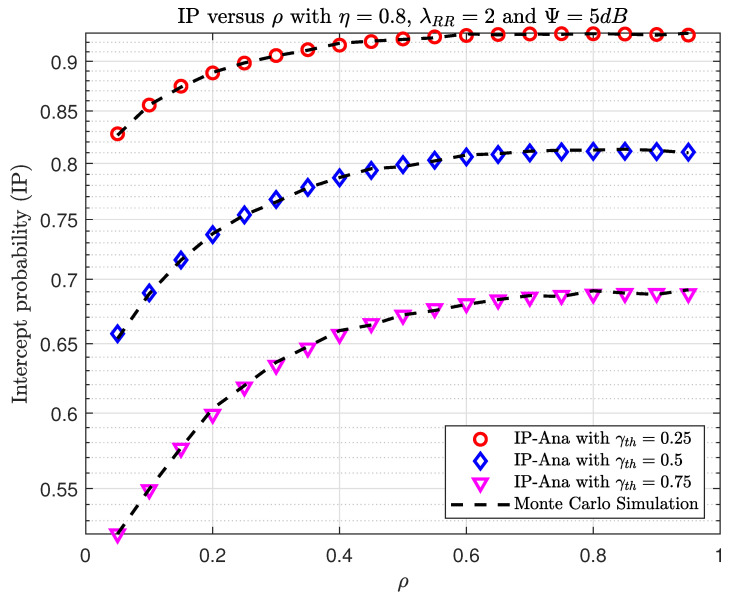
The IP versus ρ when varying $\gamma_{th}$ with η=0.8, $\lambda_{RR}=2$, and $\Psi_{th}=5$(dB).

Figure 10 and Figure 11 show the OP and IP versus ρ with different $\gamma_{th}$, respectively. The ρ value is significant since it determines not only the quantity of gathered energy at the relay but also the data transfer. First, we can observe in Figure 10 that increasing the target data required leads to an increase in OP. Second, when 0.4<ρ<0.5, the system achieves the best OP performance. In addition, when ρ increases the interception, performance increases, and when increasing $\gamma_{th}$, this will decrease the interception performance, similar to Figure 5.

## 7. Conclusions

We investigated the security and reliability of SWIPT-assistance and self-energy recycling in an AF-FD relay network consisting of an EH relay and a destination in the presence of an eavesdropper. We also evaluated the performance of the security–reliability trade-offs in terms of the OP and IP. Furthermore, Monte Carlo simulation was utilized to verify and examine the influence of the system settings on network performance, as well as the accuracy of the analytical formulations. The OP/IP asymptotic analysis was also performed to offer some insight into the system characteristics. Deep learning was developed as a novel method for predicting the system’s OP and IP with minimal computing complexity and good accuracy, which has not been investigated previously. The numerical findings demonstrated that when utilizing DNN prediction, the OP and IP outcomes were almost identical to the Monte-Carlo simulation and analysis results. As a result, deploying a DNN as a black box might be viewed as a potentially promising and effective technique for evaluating system performances via a low-latency inference procedure that avoids the derivation of complicated closed-form expressions in actual network contexts.

## Figures and Tables

**Table 1:** Comparison of our work with the related work.

	Our Work	[47]	[48]	[49]	[50]	[51]
Co-operative AF relaying network	√	√	√	√	√	√
FD	√				√	
EH	√			√	√	
PLS	√	√	√	√		√
DNN	√					

**Table 2:** Main Parameters.

Notation	Definition
$P_{S}$	The transmit power at S
$P_{R}$	The transmit power at R
$x_{S}$	The transmit signal at S with $E\left\{x_{S}^{2}\right\}=P_{S}$
$x_{R}$	The transmit signal at R with $E\left\{x_{R}^{2}\right\}=P_{R}$
η	The conversion efficiency with 0<η⩽1
ρ	The PS ratio with 0<ρ<1
$R_{\mathrm{th}}$	The target rate
$n_{R}$, $n_{D}^{1},n_{D}^{2}$, $n_{E}^{1}$, $n_{E}^{2}$	The AWGN with variance $N_{0}$
ω	The path loss exponent
$d_{\mathrm{SD}}$	The distance from S to D
$d_{\mathrm{SR}}$	The distance from S to R
$d_{\mathrm{SE}}$	The distance from S to E
$d_{\mathrm{RD}}$	The distance from R to D
$d_{\mathrm{RE}}$	The distance from R to E
$E\left\{•\right\}$	The expectation operator
$K_{\nu }\left(•\right)$	The modified Bessel function of the second kind with ν-th order:

**Table 3:** Theparameters for DNN training and testing.

Input	Value	Input	Value
ω	2	$\lambda_{\mathrm{RR}}$	[2,4]
$d_{\mathrm{SD}}$	1.5	η	0.8
$d_{\mathrm{SR}}$	1	ρ	0.25
$d_{\mathrm{RD}}$	0.5	γ	[0.5,1]
$d_{\mathrm{RE}}$	1	Ψ	[−5,25]

## Data Availability

Not applicable.
